# Expression of CD80 and CD86 costimulatory molecules are potential markers for better survival in nasopharyngeal carcinoma

**DOI:** 10.1186/1471-2407-7-88

**Published:** 2007-05-24

**Authors:** Cheng-Shyong Chang, Julia H Chang, Nicholas C Hsu, Hsuan-Yu Lin, Chih-Yuan Chung

**Affiliations:** 1 Division of Hematology-Oncology, Department of Internal Medicine, Changhua Christian Hospital, Changhua, Taiwan; 2 Department of Pathology, Changhua Christian Hospital, Changhua, Taiwan; 3 Department of Medical Education and Research, Changhua Christian Hospital, Changhua, Taiwan

## Abstract

**Background:**

B7 Costimulatory signal is essential to trigger T-cell activation upon the recognition of tumor antigens. This study examined the expression of B7-1 (CD80) and B7-2 (CD86) costimulatory molecules along with HLA-DR and the presence of infiltrating lymphocytes and dendritic cells to assess their significance in patients with nasopharyngeal carcinoma (NPC).

**Methods:**

Expression of CD80, CD86, HLA-DR, S-100 protein and the presence of infiltrating lymphocytes and follicular dendritic reticulum cells were immunohistochemically examined on the paraffin-embedded tissue blocks from newly diagnosed NPC patients (n = 50). The results were correlated with clinical outcome of patients.

**Results:**

CD80 and CD86 were each expressed in 10 of 50 cases in which they co-expressed in 9 cases. Univariate analysis revealed that patients with CD80/CD86 expression had significantly better overall survival than those without it (P = 0.017), but after adjustment for stage, nodal status, and treatment, the expression of CD80/CD86 did not significantly correlate with overall survival. Expression of HLA-DR and the presence of infiltrating lymphocytes and dendritic cells did not appear to have impact on the survival of patients.

**Conclusion:**

Expression of CD80 and CD86 costimulatory molecules appears to be a marker of better survival in patient with NPC.

## Background

Nasopharyngeal carcinoma (NPC) is a neoplasm with high incidence in Southeast Asia, the Mediterranean basin, and North Africa. Early-stage NPC is usually treated with radiotherapy alone, and combination of chemotherapy is effective in the more advanced-stages of NPC [1-4]. Identification of a pathobiological correlate of clinical behavior of NPC represents a challenge. The development of such a test may improve the outcome of treatment as high-risk patients could benefit from early intervention and aggressive treatment.

B7 Costimulatory molecules are membrane-bound molecules which play a decisive role in the activation of T cells. This costimulatory pathway involves the interaction of two distinct B7 molecules, B7-1 (CD80) and B7-2 (CD86), which are transmembrane glycoprotein members of the Ig superfamily [5-7] expressed on antigen-presenting cells with their T cell counter receptors CD28 and CTLA-4 [8]. Interaction of B7:CD28 has been shown to provide a critical signal for T cell activation, while the absence of this signal results in T cell anergy [8,9]. It is speculated that T cell anergy as the result of a paucity of B7 costimulatory molecules, which may permit the immune evasion of the cancer, is one of the mechanisms responsible for the poor immunogenicity of tumor cells. Experimental evidence has confirmed the expression of B7 costimulatory molecules in tumor cells of NPC [10,11], it is therefore interesting to determine whether B7 costimulatory molecules are factors influencing the survival outcome of patients.

In the present study, we analyzed 50 NPC tissue samples to investigate the presence of CD80 and CD86 costimulatory molecules and establish their clinical significance in NPC. The significance of infiltrating cells (lymphocytic and dendritic cells) and HLA-DR expression in tumor cells were also examined in parallel and correlated with survival in patients with NPC.

## Methods

### Patient selection and tissue specimens

Paraffin embedded tissue blocks (5–7 μm) from biopsies of newly diagnosed NPC patients between January 2001 and December 2002 were retrieved from the Department of Pathology, Changhua Christian Hospital, Taiwan (n = 50) with informed consent according to guidelines of the Changhua Christian Hospital Institution Review Board. All patients were staged according to the 1997 American Joint Committee on Cancer tumor-node-metastasis staging systems [12]. The pathological stage and nodal status were obtained from the primary pathology reports. Slides from tumors were reviewed by two pathologists to define the histological grading. The survival data were either obtained from the cancer registry of Changhua Christian Hospital or collected from the patients' attending physicians. 42 of the 50 patients received concurrent chemoradiotherapy consisting of cisplatin and fluorouracil. Five patients (T1-2 N0) along with three patients with T stage of 3–4 refused chemotherapy were treated with radiation alone. The median follow-up period was 56 months (range, 1–73 months).

### Immunohistochemical staining

Blocks were sectioned and put on poly-1-lysine coated slides. After deparaffinized, the section was treated with 3% H_2_O_2 _in methanol. The sections were then hydrated through gradient alcohol and PBS. Slides were placed in 10 mM citrate buffer and heated for 20 minutes in a 700-W microwave oven at 100°C in PBS. Slides from each case were exposed to 1:200 dilution of anti-CD80 monoclonal antibody (Santa Cruz Biotechnology, USA) and 1:100 dilution of anti-CD86 polyclonal antibody (Immunotech, Marseille, France) for 30 minutes at room temperature followed by incubation with a PicTure Polymer Kits for 20 minutes (Zymed, South San Francisco, USA). The sections were thoroughly washed with PBS between steps. The sites of peroxidase were visualized with 3,3'-diaminobenzidine tetrahydrochloride. Hematoxylin was used for counterstaining. The percentage of immunoreactivity in the tumor cells was scored as 0 to +2. Immunoreactivity in <10% of tumor cells was considered as aberrant expression (-), 10–50% (+1), and >50% (+2). All fields in the sections were examined. Investigator-bias was avoided by two investigators independently scoring coded sections. Furthermore, CD80 and CD86 positive samples were repeated to ensure that no false positive results occurred in the experiment.

Same steps and scoring systems were carried out with the following monoclonal and polyclonal antibodies to address the expression of HLA-DR and the presence of infiltrating lymphocytes and dendritic cells: anti-CD45R0, anti-CD20, and anti-Follicular Dendritic Reticulum Cells (all from Dakocytomation, Denmark); anti-HLA Class II (DR) and anti-S-100 (both from Novocastra Laboratories, UK).

### Statistical analysis

Survival curves in accordance with various potential prognostic indicators were produced. Survival was defined as the time between date of diagnosis and date of death. Patients still alive at the end of the study were censored at the date of last follow-up. Sections scored as +1 and +2 were identified as positive staining to decrease the number of variables for statistical purpose. Survival rate was computed using standard Kaplan-Meier methods, and the difference in survival curves was analyzed by the log-rank test. Independent prognostic factors were analyzed by the Cox proportional hazards regression model. Variables in the model included T stage, nodal (N) stage, and treatment (radiotherapy or concurrent chemoradiotherapy). A P value of less than 0.05 was considered to indicate statistical significance.

## Results and discussion

### CD80/CD86 expression

**Figure 1:**
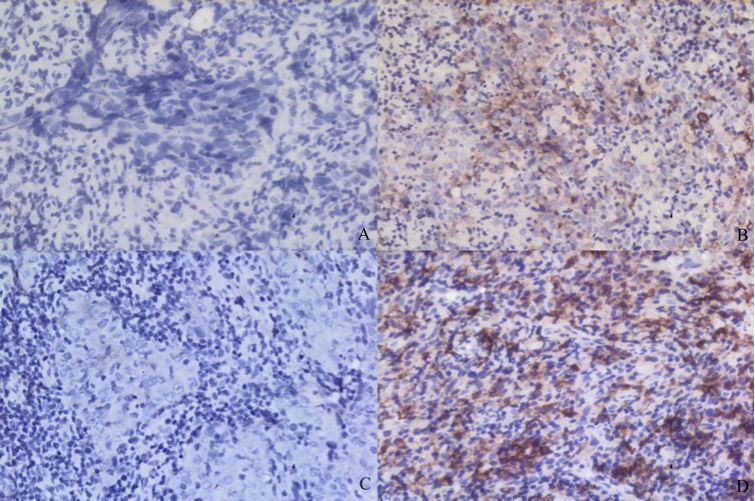
Immunohistochemical staining for (A) CD 80^- ^and (B) CD80 (+1) in undifferentiated carcinoma of the nasopharynx (×400); (C) CD 86^- ^in non-keratinizing squamous cell and (D) CD86 (+2) in undifferentiated carcinoma of the nasopharynx (×400).

Regardless of stage and histological type, CD80 and CD86 were each expressed in 10 of 50 cases in which they co-expressed in 9 cases. Expression of CD80 and CD86 were classified as a group to reduce the number of prognostic variables as 9 patients had overlapping expression. Positive membrane and occasional cytoplasmic staining of CD80/CD86 was observed in 11 of the 50 patients (Figure 1, Table 1). CD80/CD86 expression was not noticed in normal nasopharyngeal mucosal epithelia. Abnormal nasopharyngeal epithelia were hardly present in our biopsies.

### Uni-and multivariate analyses for prognostic factors

**Figure 2:**
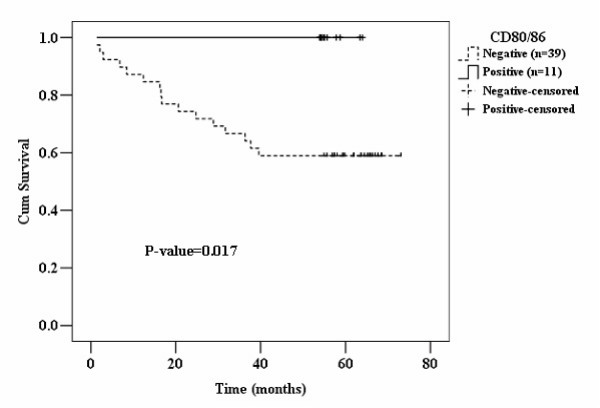
Kaplan-Meier survival curves based on expression of CD80/CD86 for patients with nasopharyngeal carcinoma.

The Univariate analysis revealed that there was a significant association between expression of either CD80 or CD86 and clinical outcome in terms of survival (P = 0.017) (Table 2). The Kaplan-Meier survival curves of the 50 NPC patients were plotted into 2 groups according to immunostaining results with either CD80/CD86 positive or negative (Figure 2). Due to the limited number of patients, in the multivariate analysis which incorporated independent prognostic factors of T stage, N stage, and treatment (concurrent chemoradiotherapy vs. radiotherapy alone), we found that CD80/86 expression was not significant with regard to survival (Table 3). No association was observed between CD80/86 expression and the infiltration of T-cells and dendritic cells (Table 4).

### T, B, and dendritic cell infiltration and HLA-DR expression in NPC tissue sections

**Figure 3:**
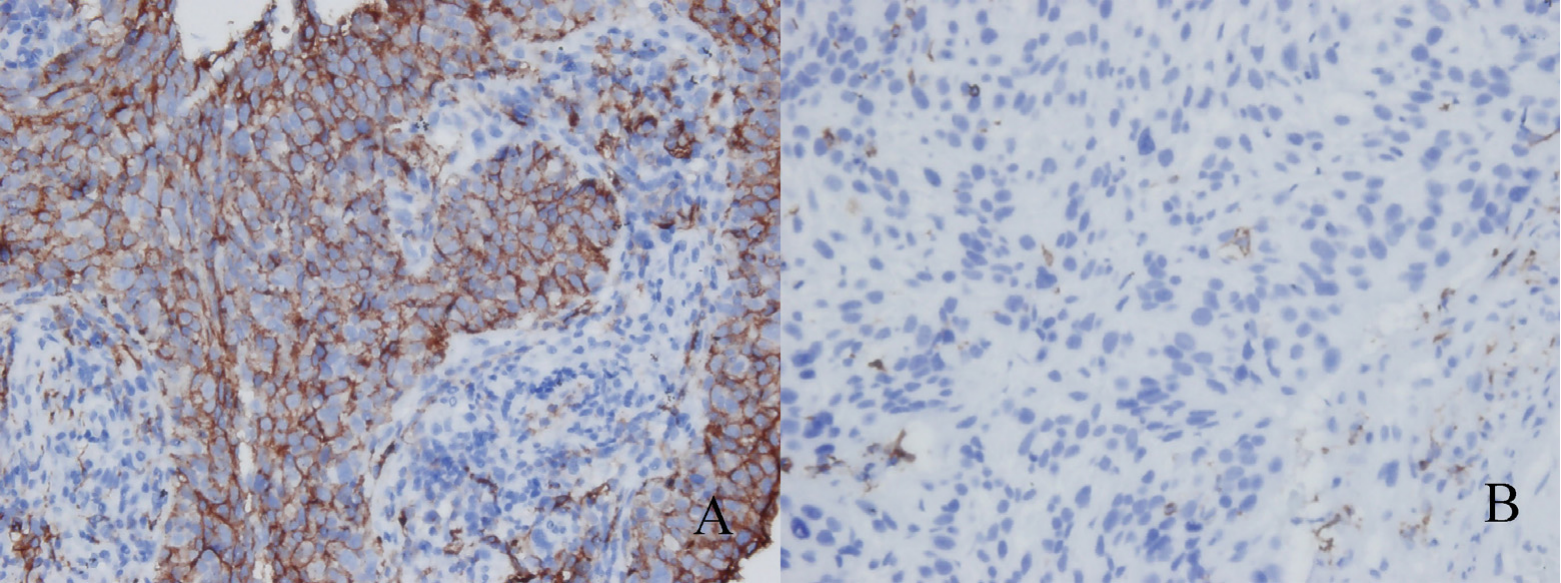
Positive membrane staining of HLA-DR in undifferentiated carcinoma, 400 × (A); negative staining of HLA-DR in carcinoma cellsand dispersed lymphocytes with HLA-DR expression, 400 × (B).

Subpopulations of infiltrating lymphocytes in NPC were identified using anti-T (CD45R0) and anti-B (CD20) cell antibodies. Follicular dendritic reticulum cells, S-100 protein which marks infiltrating dendritic cells, along with tumor HLA-DR (Figure 3) expression were also identified in this study. However, none were statistically significant for survival (Table 2).

## Conclusion

Studies have described the presence of B7 costimulatory molecules in tumor cells of NPC, and the corresponding counter-receptor, CD28, in lymphoid cells, implying some communication between T cells and carcinoma cells in the microenvironment of NPC [10,11]. These data suggest certain level of immune response directed at the NPC tumor cells with the presence of B7 costimulatory molecules in tumor cells of NPC and the vast number of infiltrating T cells. This phenomenon might explain why in the majority of NPC patients, the large prevalence of T cells in the neoplastic tissue does not have any prognostic significance. The presence of B7 costimulatory molecules is essential for the activation of T cells.

Our experimental evidence supports the finding that in a subset of NPC patients, tumor cells indeed express B7 costimulatory molecules. CD80 and CD86 were each expressed in 10 of 50 of our NPC cases in which they co-expressed in 9 cases. Due to the limited number of NPC cases, statistical conclusions which correlate the expression of CD80/86 with survival can not be drawn in the multivariate analysis in this study. Additional studies with larger number of patients are needed to confirm this finding.

However, this study demonstrated the presence of B7 costimulatory molecules in the neoplastic tissue of NPC and their possible prognostic importance. It is the first to show a significant association between expression of CD80/CD86 and a favorable outcome in patients with NPC by univariate analysis.

With a relatively small group of patients, our data suggested that neither expression of HLA-DR, the presence of infiltrating dendritic cells (follicular and S-100(+) interdigitating), nor the prevalence of lymphocytes in the neoplastic tissue had any prognostic significance for survival in patients with NPC. However, we can not rule out that with large number of patients, the prognostic factors might have been significant on the basis of this report.

The absence or low level expression of B7 costimulatory molecules by malignant cells is thought to represent one mechanism by which they escape immunosurveillance. Recently, CD80 expression has been demonstrated to be closely associated with decreased tumorigenicity in oral squamous carcinoma, suggesting that inadequate CD80 expression during early oral squamous cell carcinoma formation may contribute to the escape of tumors from the immune system [13]. The fact that 9 of the 11 (82%) CD80/86 positive patients were in T stage of 1–2 provide indirect evidence that expression of CD80/86 may serve as a marker for decreased tumorigenicity during early development of NPC.

The question of whether expression of B7 costimulatory molecules represent enhanced immunity against tumor has important implications with regard to the prognosis and treatment of NPC. The demonstration in this study which addresses the presence of B7 costimulatory molecules in the neoplastic tissue of NPC and their possible role in the prognosis of this disease is important. It should be noted that B7 expression could be widely determined on routinely processed, paraffin-embedded tissues for prognostic evaluation in NPC cases, whereas the quantification of plasma EBV DNA [14] can only be reliably performed for the purpose of clinical evaluation in sophisticated centers. Finally, integration of multiple markers and tools through use of statistical modeling should improve our ability to identify high-risk patients who may benefit from refined treatment strategies.

## Abbreviations

NPC – nasopharyngeal carcinoma

EBV – Epstein-Barr virus

## Competing interests

The author(s) declare that they have no competing interests.

## Authors' contributions

CSC designed the study and drafted the manuscript. JHC carried out the immunohistochemical staining. NCH participated in the statistical analysis. HYL helped to design the study. CYC participated in the coordination of the study. All authors read and approved the final manuscript.

## Pre-publication history

The pre-publication history for this paper can be accessed here:



## Figures and Tables

**Table 1:** Characteristics of 50 patients with nasopharyngeal carcinoma

**Characteristics**		Overall		CD80/86(+)	
		No.	%	No.	%

**Age, years**					
	Range	38–83		38–78	
	Median	50		46	
	Mean	53		50	
**Sex**					
	Male	42	84	10	91
	Female	8	16	1	9
**Pathology (WHO classification)**					
	Type I	--		--	
	Type II	35	70	6	55
	Type III	15	30	5	45
**T Stage (1997 AJCC)**					
	T1 or T2	32	64	9	82
	T3 or T4	18	36	2	18
**N Stage (1997AJCC)**					
	N0 or N1	24	48	6	55
	N2	17	34	3	27
	N3	9	18	2	18
**Overall Stage (1997 AJCC)**					
	I	5	10	3	27
	II	10	20	3	27
	III	13	26	2	18
	IV	22	44	3	27
**Treatment**					
	Radiotherapy alone	8	16	2	18
	Concurrent Chemoradiotherapy	42	84	9	82
**Status**					
	Death	16	32	0	0
	Alive	34	68	11	100

Abbreviations: WHO, World Health Organization; AJCC, American Joint Committee on Cancer; FDRC, Follicular Dendritic Reticulum Cells

**Table 2:** Univariate analysis for overall survival of patients with nasopharyngeal carcinoma

Kaplan-Meier Survival Rate						
				Censored		
					
		Total	Dead	N	%	P-value

	Overall	50	16	34	68.0	
						
T	Negative	7	3	4	57.1	0.543
	Positive	43	13	30	69.8	
						
B	Negative	9	4	5	55.6	0.253
	Positive	41	12	29	70.7	
						
FDC	Negative	20	8	12	60.0	0.286
	Positive	30	8	22	73.3	
						
CD80*	Negative	40	16	24	60.0	0.025
	Positive	10	0	10	100.0	
						
CD86*	Negative	40	16	24	60.0	0.025
	Positive	10	0	10	100.0	
						
CD80/86*	Negative	39	16	23	59.0	0.017
	Positive	11	0	11	100.0	
						
HLA1DR	Negative	15	7	8	53.3	0.167
	Positive	35	9	26	74.3	
						
S100	Negative	20	7	13	65.0	0.654
	Positive	30	9	21	70.0	

P-value by Log-rank test

Follow-up time: from diagnosis date till death/2-15-2007

**Table 3:** Multivariate analysis of clinicopathologic factors for overall survival of patients with nasopharyngeal carcinoma

		Total	Dead	%	HR	95% C.I. for HR			P-value
CD80/86	Negative	39	16	41.0	1.000				0.968
	Positive	11	0	0.0	0.000	0.000	-	6.853E+282	
									
T Stage	1 or 2	32	7	21.9	1.000				0.050
	3 or 4	18	9	50.0	2.892	1.001	-	8.353	
									
N Stage	0 or 1	24	5	20.8	1.000				0.065
	2 or 3	26	11	42.3	2.830	0.938	-	8.539	
									
Treatment	RT	8	1	12.5	1.000				0.193
	CCRT	42	15	35.7	3.979	0.497	-	31.870	

HR: Hazard Ratio

**Table 4:** Association of CD80/86 expression and infiltration of T-cell/Dendritic cells in nasopharyngeal carcinoma

		CD80/86							
						
		Negative (n = 39)		Positive (n = 11)		Total (n = 50)		Φ	P-value
					
		N	%	N	%	N	%		
T	Negative	6	15.4	1	9.1	7	14.0	0.075	1.000
	Positive	33	84.6	10	90.9	43	86.0		
									
FDC	Negative	13	33.3	7	63.6	20	40.0	-0.256	0.090
	Positive	26	66.7	4	36.4	30	60.0		
									
S-100	Negative	16	41.0	4	36.4	20	40.0	0.039	1.000
	Positive	23	59.0	7	63.6	30	60.0		

P-value by Fisher's Exact Test

Φ: Phi correlation coefficient
